# Performance of the MOLES and TFSOM-DIM scores in classifying choroidal nevi and melanoma

**DOI:** 10.1038/s41598-024-78692-w

**Published:** 2024-11-18

**Authors:** David Jahnke, Carsten Grohmann, Bettina Fuisting, Christos Skevas, Martin S. Spitzer, Johannes Birtel

**Affiliations:** 1 Department of Ophthalmology, University Medical Center Hamburg-Eppendorf, Hamburg, Germany; 2 Department of Ophthalmology, University of Bonn, Bonn, Germany

**Keywords:** Choroidal nevus, Choroidal melanoma, Retinal imaging, ocular oncology, imaging, Medical research, Eye manifestations

## Abstract

Choroidal nevi are common benign melanocytic lesions often incidentally found during routine ophthalmic examinations. Patients with choroidal nevi are frequently referred to ocular oncology centers to differentiate these from choroidal melanoma and to exclude a malignant transformation which rarely occurs. However, this causes a high workload for referral centers and may delay diagnosis and treatment of patients with potentially severe conditions. To overcome this, the MOLES score was previously developed which may empower non-specialists to differentiate choroidal nevi from melanoma. In this retrospective study, the performance of the MOLES score was assessed in 695 patients with choroidal nevi and 53 with choroidal melanoma. With a specificity of 96% (choroidal nevi) and a sensitivity of 100% (melanoma) the MOLES score was highly successful in differentiating choroidal nevi from melanoma and may offer great potential to optimize monitoring and referral decisions, and for teleophthalmology applications. Furthermore, the TFSOM-DIM score using risk factors for determining tumor progression was applied and showed a good correlation with MOLES. A complementary use of MOLES and TFSOM-DIM may be beneficial for clinical routine: MOLES for screening by non-experts with limited diagnostic tools and TFSOM-DIM in a referral setting to counsel also regarding potential transformation.

**Supplementary Information:**

The online version contains supplementary material available at 10.1038/s41598-024-78692-w.

## Introduction

Choroidal nevi are common benign melanocytic lesions with an estimated prevalence of about 5–8% in Caucasians^1,2^. The rate of malignant transformation into choroidal melanoma is rare with about 5 cases per million per year^3^. As choroidal lesions are often found incidentally during routine examinations, patients with benign lesions are commonly referred to ocular oncology centers for diagnosis and monitoring. This puts pressure on these centers, causes costs for the healthcare system as well as for patients, and may delay diagnosis and treatment of patients with potentially severe conditions^4,5^.

An early and accurate diagnosis of choroidal melanoma and a precise differentiation to choroidal nevi is vital to achieve timely treatment and better therapeutic outcomes. The distinction between choroidal nevi and melanomas may sometimes be difficult. For instance, indeterminant melanocytic lesions^6^ may present as small choroidal melanomas which may be misdiagnosed as nevi or large nevi that may imitate melanoma. To differentiate choroidal nevi from melanomas as well as to assess the risk of malignant transformation, several mnemonics have been developed.

One of these systems is the MOLES score which was developed to empower non-specialists to differentiate choroidal nevi from melanoma and to optimize monitoring and referral decisions for melanocytic choroidal tumors^7^. To estimate the risk of malignancy, 5 features of choroidal melanoma are assessed by retinal imaging mainly. Based on the score, the MOLES guideline recommends referral pathways and follow-up-intervals.

This study aimed to analyze the effectiveness of the MOLES score in a German tertiary referral center and contrasted the results of the MOLES score with the TFSOM-DIM score.

## Methods

This retrospective study was performed at the Department of Ophthalmology at the University Medical Center Hamburg-Eppendorf, Germany and was in adherence to the Declaration of Helsinki. All clinical investigations and diagnostic genetic testing were performed as part of routine clinical care. Due to the retrospective nature of the study, the ethics committee of the Hamburg Medical Association waived the need of obtaining informed consent.

### Patients

Patients were identified with a local audit of the electronic patient records. Using a Python script, patient records with the keywords “melanom”, “nävus” or “nevus” were identified. Localization of tumor (temporal, nasal, superior, inferior from the fovea; macular region), laterality, tumor characteristics, sex, the MOLES- and TFSOM-DIM scores were assessed for each patient. If a patient had bilateral assessable nevi, only the right eye was selected for analysis. Eyes without comprehensive imaging or poor imaging quality were excluded.

### Retinal imaging

Retinal imaging included wide-field color fundus photography, fundus autofluorescence, fluorescence angiography (all, Optos, Dunfermline, UK), optical coherence tomography (Spectralis, Heidelberg Engineering, Heidelberg, Germany), Topcon OCT (DRI OCT Triton, Topcon, Tokyo, Japan), and B-scan ultrasonography (ABSolu, Quantel, Lumibird Medical Cournon d’Auvergne Cedex, France)^8^.

### MOLES score

The MOLES acronym stands for Mushroom shape, Orange pigment, Large size (≥ 3 disc diameters or thickness ≥ 1 mm), Enlarging tumor and Subretinal fluid (SRF). Fundus photography and fundus autofluorescence (AF) were used to determine orange pigment, tumor diameter (using the optic disc as reference), enlargement as well as visible subretinal fluid or retinal detachment. Enlargement was determined by analyzing tumor boundaries relative to adjacent landmarks, as recommended^9^. OCT was used to assess subretinal fluid, tumor thickness and mushroom shape. B-scan ultrasonography was used to determine mushroom shape, tumor thickness and acoustic density.

**Fig. 1:**
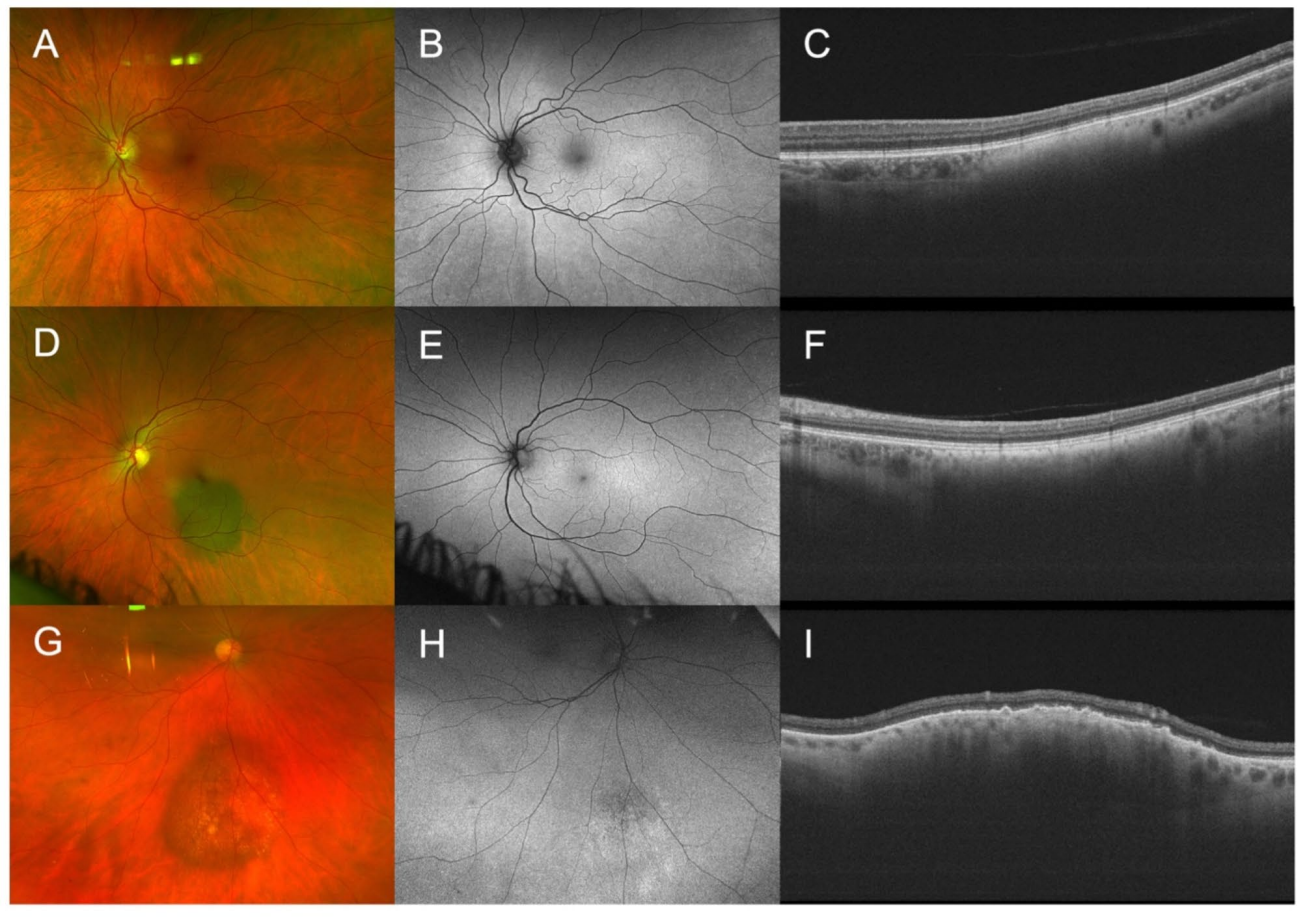
Examples for nevi assessed using MOLES. (**A**-**C**): “Common nevus” (MOLES 00000) with a basal diameter of 2DD. (**D**-**F**): “Low risk nevus“ (MOLES 00100) with a basal diameter of 3DD. (**G**-** I**): “High risk nevus” (MOLES 00200) with a basal diameter of 4.5DD and prominence on OCT. From left to right: fundus photography, fundus autofluorescence, OCT imaging.

Each feature was assessed and scored with either 0 points if absent, 1 point if unsure or borderline present, and 2 points if distinctly present (Supplementary Table 1). All scores were then added up to the total score. A total score of 0, 1, 2, ≥ 3 was categorized as “common nevus” (Fig. 1A-C), “low-risk nevus” (Fig. 1D-F), “high-risk nevus” (Fig. 1G-I) and “probable melanoma”, respectively. The MOLES referral guidelines recommend self-check up in the community every two years for “common nevi”, non-urgent referral to specialists with imaging every 6 to 12 months for “low-” and “high-risk nevi” and urgent referral for “probable melanoma” (Supplementary Table 2).

### TFSOM-DIM score

The TFSOM-DIM score was developed to determine the 5-year growth rate of choroidal nevi into melanoma based on six risk factors. These include tumor thickness > 2 mm (based on ultrasound), subretinal fluid (based on OCT), symptoms of vision loss ≥ 20/50 (Snellen chart), orange pigment (using AF), melanoma acoustic hollowness (based on ultrasound) and tumor diameter > 5 mm (using fundus photography)^10^. In contrast to MOLES, TFSOM-DIM is a binary scoring system, attributing 0 points to the feature if absent, or 1 point if present (Supplementary Table 3). With an increase of risk factors, the total score and consecutive growth rate is higher. A lesion with 0 risk factors has a 5-year growth rate of about 1%, while a lesion with 5 risk factors has a rate of about 55%^10^.

## Results

In total, 1746 patients with choroidal nevi and 122 with choroidal melanoma were identified. Out of those, 1051 patients with nevi and 69 with melanoma had to be excluded due to lack of comprehensive imaging or poor imaging quality. Thus, the study cohort included 695 patients (61% females; 51% right eyes) with nevi and 53 (45% females; 51% right eyes) with melanomas. Of the nevi, 124 (18%) were in the superior, 180 (26%) in the nasal, 105 (15%) in the inferior, 208 (30%) in the temporal quadrant, and 78 (11%) in the macular region. Of the melanomas, 7 (13%) were in the superior, 15 (28%) in the nasal, 10 (19%) in the inferior, 19 (36%) in the temporal quadrant, and 1 (2%) in the macular region. The location of one melanoma could not be specified due to extensive growth.

### MOLES scores of nevi

The MOLES scores for the nevi were 0, 1, 2 and ≥ 3 in 444 (64%), 144 (21%), 81 (12%), and 26 (4%), respectively. The most common feature present (2 points) or borderline present (1 point) were large size (26%), enlargement (8%), orange pigment (6%), and SRF (5%) (Supplementary Table 4). The specificity to detect choroidal nevi as benign was 96% with a mean MOLES score of 0.57.

**Fig. 2:**
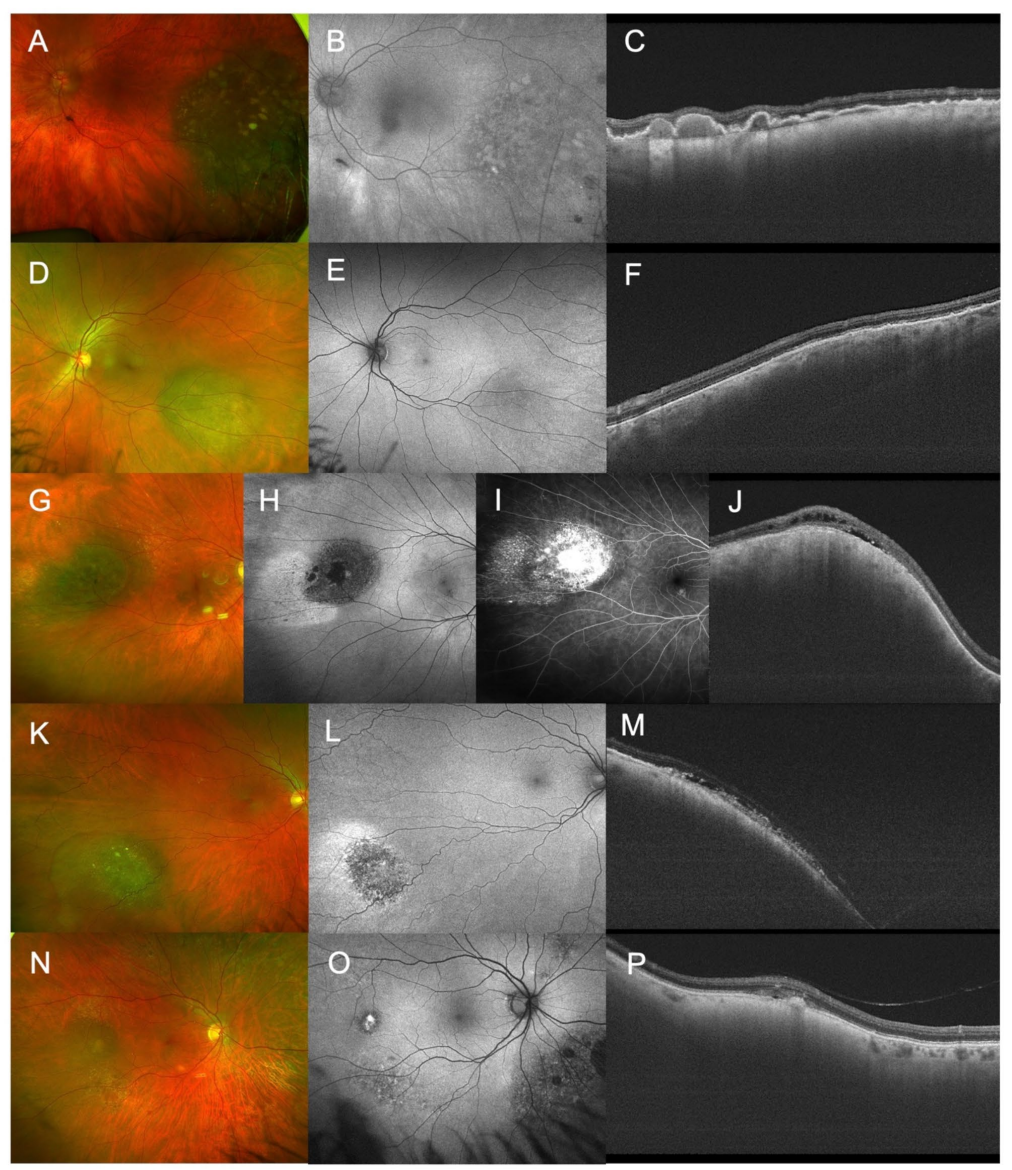
Examples of high-risk nevi labelled as probable melanoma. (**A**-**F**): Fundus photography, fundus autofluorescence and OCT of giant nevi with a basal diameter > 5 (MOLES 00220). (**G**-**J**): Fundus photography, fundus autofluorescence, fluorescence angiography and OCT of a large nevus with orange dusting and SRF (MOLES 01101). (**K**-**M**): Fundus photography, fundus autofluorescence and OCT of a large nevus with orange dusting and SRF (MOLES 01201). (**N**-**P**): Fundus photography, fundus autofluorescence and OCT of a small nevus with orange clumps and trace SRF (MOLES 02001).

Nevi with a MOLES score ≥ 3 (“probable melanoma”) included patients with 3 (*n* = 14) and 4 points (*n* = 12) and commonly had the characteristics large size and/or enlargement. Nine of these, had a diameter of > 5 disc diameters (DD) and/or thickness > 3 mm leading to a MOLES score of 00220 (score 4) (Fig. 2). 24 of these lesions were monitored closely; for the remaining two transpupillary thermotherapy and proton therapy were considered, but not performed.

### MOLES scores of nevi transformed into melanoma

**Fig. 3:**
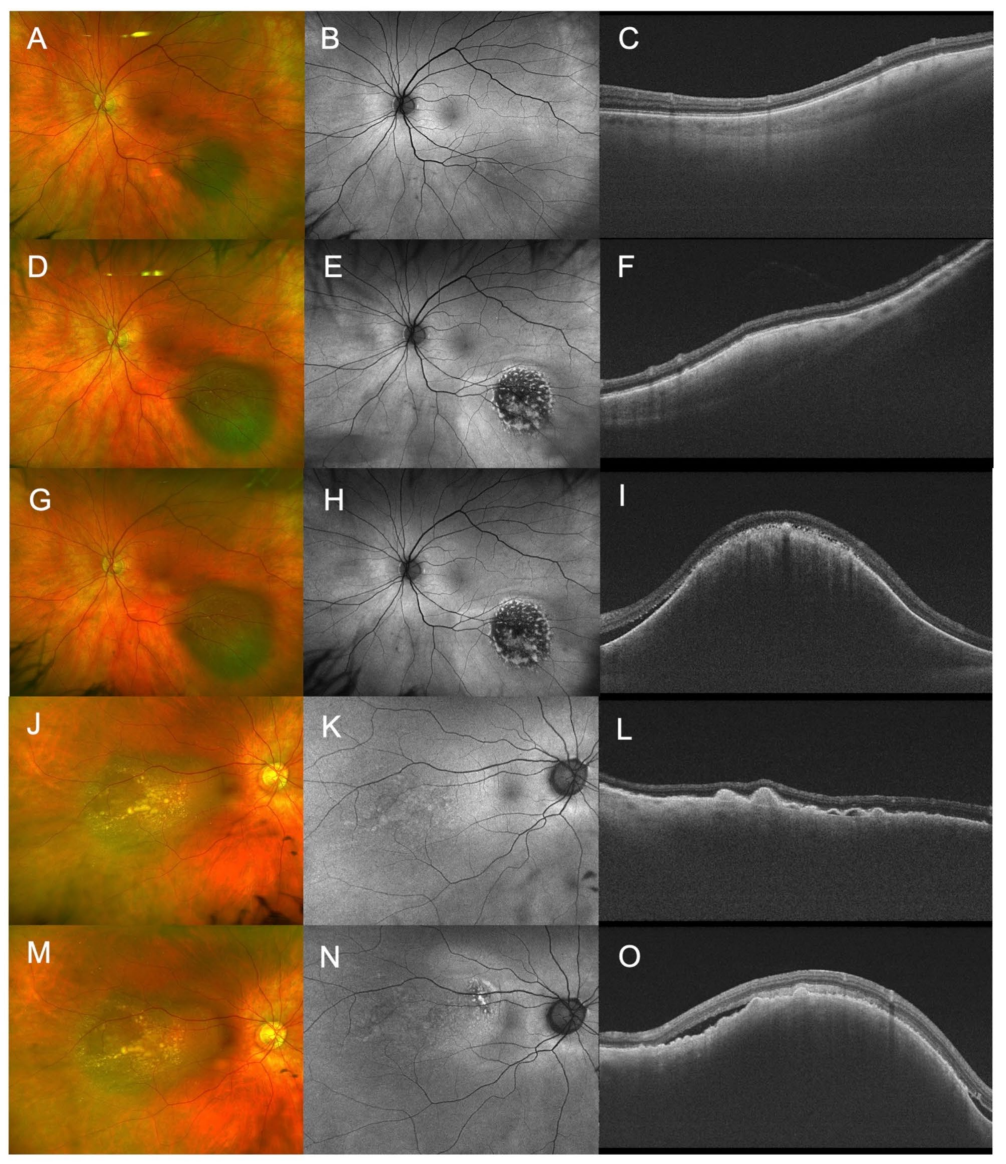
Two cases of malignant transformation. (**A**-**C**): Multimodal imaging of a choroidal nevus (MOLES 00100) which transformed to a choroidal tumor; (**D**-**F**) one year after first presentation growth in basal diameter and orange clumping were observed (MOLES 02220); (**G**-**I**) further growth in thickness and SRF were observed before treatment with proton therapy (MOLES 02221). (**J**-**L**): Imaging of a large choroidal nevus (MOLES 00200) which progressed within 6 months and showed at follow up (**M**-**O**) confluent orange pigment and SRF (MOLES 02201). From left to right: fundus photography, fundus autofluorescence, OCT imaging.

Sequential imaging was available for 368 nevi. Over the observation period (median: 17 months; range 6-131 months), 7 nevi transformed into choroidal melanoma. Out of those, 5 had an initial MOLES score below 3 and demonstrated at follow up a score ≥ 3. This included an intermediately large nevus with SRF developing orange pigment; a large nevus developing orange clumps and SRF (Fig. 3); a large nevus developing extensive retinal detachment; a nevus increasing in size and developing SRF; and an initially small nevus increasing in size, developing orange clumps, and SRF (Fig. 3). All these tumors were treated shortly after transformation. In two cases, tumors clinically classified as nevi / indeterminant lesions had a MOLES score of ≥ 3. Both were closely followed up and underwent treatment 18 and 6 months, respectively, after initial presentation.

### MOLES scores of melanoma

The MOLES scores for the clinically diagnosed melanoma (*n* = 53) were ≥ 3 in all cases: A MOLES score of 3, 4, 5, 6, 7, 8, 9, 10 was revealed in 12 (23%), 8 (15%), 8 (15%), 9 (17%), 4 (8%), 6 (11%), 5 melanomas (9%) and 1 melanoma (2%), respectively. This results in a sensitivity of 100%; the mean MOLES score was 5.5.

The most common feature present (2 points) or borderline pronounced (1 point) were large size (98%), SRF (87%), enlargement (75%), orange pigment (60%), and mushroom shape (23%) (Supplementary Table 5).

### TFSOM-DIM scores of nevi

The TFSOM-DIM score was assessed in 211 patients with nevi and was 0, 1, 2, 3 and 4 for 106 (50%), 67 (32%), 30 (14%), 7 nevi (3%) and 1 nevus (1%), respectively. The TFSOM-DIM scores increased with a higher MOLES score. The mean TFSOM-DIM score was 0.14 for a MOLES of 0; 0.6 for MOLES 1; 1.43 for MOLES 2; to 1.95 for MOLES ≥ 3. The most common features detected were a diameter ≥ 5 mm (31%), symptomatic vision loss (15%), orange pigment (10%), SRF (8%), low acoustic internal reflectivity (6%), and thickness > 2 mm (1%).

### TFSOM-DIM scores of nevi transformed into melanoma

5 out of the 7 nevi that transformed into melanoma had analyzable ultrasonography images available to which the TFSOM-DIM score was applied. This included a nevus without any TFSOM-DIM risk factors at initial presentation that increased in thickness, developed hollowness on ultrasonography, and SRF; a nevus with SRF developed hollowness on ultrasonography and orange pigment; a nevus with large diameter developed hollowness, orange pigment, SRF, and growth in thickness; and a large nevus with SRF and low internal acoustic reflectivity developed reduced visual acuity and orange pigment.

### TFSOM-DIM scores of melanoma

The distribution of TFSOM-DIM scores for melanomas (*n* = 36) was 2, 3, 4, 5, 6 for 2 (6%), 9 (25%), 9 (25%), 8 (22%) and 8 melanomas (22%), respectively. The most common feature present was low acoustic internal reflectivity (92%), SRF (81%), diameter ≥ 5 mm (75%), orange pigment (69%), thickness > 2 mm (67%), and symptomatic vision loss ≤ 20/50 (47%) (Supplementary Table 6).

## Discussion

Mnemonics may play an important role in medicine. They are easy to remember and condensed tools that may help to identify and remember key features of diseases. MOLES and TFSOM-DIM are prominent mnemonics used in ocular oncology; this work applied both to a German cohort and assessed whether these may be valuable.

With a specificity of 96% (choroidal nevi) and a sensitivity of 100% (melanoma) the MOLES score was highly successful in differentiating choroidal nevi from melanoma. The high sensitivity underlines the strength of MOLES in having a low threshold for recognizing malignancy^11,12^. The amount of high-risk nevi labelled as probable melanoma will therefore be higher than melanomas categorized as nevi.

Many patients with choroidal nevi are regularly referred to ocular oncology clinics in Germany. This results in high pressure on such clinics with impacts on staff, diagnostics, and capacities for serious ocular oncology cases. Some of these challenges may be overcome by using the MOLES score which was developed to help non-experts to screen choroidal nevi. This is reflected in the fact that almost two thirds of the nevi referred to our ocular oncology clinic were “common nevi”, which may be locally followed without need of a referral^13^.

This study also showed that patients requiring urgent referral to an ocular oncologist due to signs of malignancy or nevus transformation were identified using the MOLES score. This may indicate that MOLES is a successful scoring system in real world settings. MOLES allows for moderately pronounced features to be assigned an intermediate category which may support non-experts feeling unsure if a feature is present or absent^9^. In our cohort, two initially indeterminant lesions received treatment over the disease course due to concern of malignant transformation. Reviewing these with the MOLES score, both were classified as “probable melanoma”. Instead of monitoring these tumors as indeterminant lesions, an early classification as “probable melanoma” may have been beneficial to reduce risk of tumor progression.

As the MOLES score avoids a binary score (absent vs. present) and has the option to classify lesions as “unsure” or “borderline present”, for instance if low-quality images are available or if the ophthalmologist has limited experience, this may cause some over-referrals^9,11^. Further limitations of the MOLES score include that MOLES is not suitable for detecting other choroidal tumors such as melanocytoma, congenital ocular melanocytosis, choroidal hemangioma and metastases^9^. Additionally, as a purely diagnostic tool, MOLES was not developed to provide indication for therapy in the case of choroidal melanoma^7^.

MOLES was developed to determine features with ophthalmoscopy and fundus photography only^7,14^. Therefore, a concern could be that it may have a lower reliance for detecting malignancy as ultrasonography is not included. A tumor with sole increase of thickness in ultrasonography or reduction of acoustic internal reflectivity could be missed. However, this limitation may be (partially) overcome as melanomas typically show a simultaneous increase in basal diameter and/or appearance of other signs of malignancy^15^. Al Harby et al. showed, that only around 3% of choroidal tumors had a thickness in ultrasonography which influenced (increased) the final MOLES score^12^. Roelofs et al. demonstrated that ultrasonography was relevant in detecting progression of choroidal melanocytic tumors in only 1% of cases^15^.

The MOLES score may also be valuable for tele-ophthalmology settings and potentially artificial intelligence^16,17^. Digital screening programs with tumor grading may support local eyecare providers especially for “common nevi”. This may strengthen those practices and reduce (travel) costs, the carbon footprint associated with visits, and time spent in clinic for patients^18^. Digital assessment of choroidal nevi has gained relevance in the UK since the COVID-19 pandemic and tele-ophthalmological screening programs are already being applied in other areas such as for diabetic retinopathy^19–21^. Similar developments may be seen in Germany and other countries. However, for such applications standardized multimodal imaging but also medical education of the local ophthalmologists appears crucial to implement the acceptability^11,22,23^.

The second tool analyzed was the well-established and recently modified TFSOM-DIM score which uses risk factors for determining tumor progression over a period of 5 years^10,24,25^. Applying the TFSOM-DIM score to a subset of 211 nevi and 36 melanomas showed an overall good correlation of TFSOM-DIM with MOLES. A higher MOLES score was associated with a higher TFSOM-DIM score. The MOLES and TFSOM-DIM scores may be used complementary to each other: MOLES for screening by non-experts that usually have limited diagnostic tools and may lack ultrasonography^15^ and TFSOM-DIM to assess patients in a referral setting including ultrasonography and to counsel patients regarding the 5-year transformation. Patients may benefit from the “precise” risk provided which might be more informative and specific than “low-” or “high-risk nevus”. However, TFSOM-DIM does not recommend follow-up intervals which may lead to differences between ocular oncology centers.

A strength of this study is the combined number of choroidal melanocytic tumors analyzed using MOLES, which is larger than in previous works^7,11,12,15^. Furthermore, this is the first study applying MOLES to a German setting with the German referral structure. The results of this study may help to extend the applicability of MOLES outside the UK^9^. Limitations of this study include the retrospective design which also led to exclusion of a high number of patients of the initial data set. Furthermore, this study included only limited follow-up intervals of nevi. Determining the rate of malignant transformation of nevi categorized as “high-risk” or “probable melanoma” over the course of multiple years would be interesting to analyze, however, this was beyond the scope of the study.

## Conclusion

This study demonstrates that the MOLES scoring system can be successfully applied to healthcare settings outside the UK as a high specificity in detecting choroidal nevi and high sensitivity in detecting choroidal melanoma may be achieved. MOLES appears as a useful screening tool and may release pressure from reference centers.

## Electronic supplementary material

Below is the link to the electronic supplementary material.

Supplementary Material 1.

## Data Availability

The datasets generated during and/or analyzed during the current study are available from the corresponding author on reasonable request.
