# Supplementary material for: Performance of the MOLES and TFSOM-DIM scores in classifying choroidal nevi and melanoma
Source: Sci Rep. 2024 Nov 18;14:28534. doi: 10.1038/s41598-024-78692-w (PMC11574176; doi:10.1038/s41598-024-78692-w)
Supplement: Supplementary file 1 — Supplementary Material 1. [file 41598_2024_78692_MOESM1_ESM.pdf]

SUPPLEMENTARY MATERIAL

Supplementary Table 1: MOLES scoring system

**Risk factor**	**M**ushroom shape	**O**range pigment	**L**arge size	**E**nlargement	**S**ubretinal fluid
0 Points	Absent	Absent	Diameter <3DD <u>and</u> Thickness <1mm	None <u>or</u> No previous ophthalmoscopy (new lesion)	Absent
1 Point	Unsure <u>or</u> Incipient	Unsure <u>or</u> Dusting	Diameter 3-4DD <u>and/or</u> Thickness 1-2mm	Unsure <u>or</u> Minimal <u>or</u> Previous Ophthalmoscopy without photography (new lesion)	Minimal (only on OCT)
2 Points	Present with overhang	Confluent clumping	Diameter >4DD <u>and/or</u> Thickness >2mm	Definite <u>or</u> Previous imaging did not show the new lesion <u>or</u> Diameter > 5DD or Thickness > 3mm	Seen on fundus photography <u>or</u> Extending beyond tumor margins

DD, disc diameter; OCT, optical coherence tomography

Adapted from: Damato BE. Can the MOLES acronym and scoring system improve the management of patients with melanocytic choroidal tumours? Eye (Lond). 2023;37(5):830-6.

Supplementary Table 2: MOLES referral guidelines for managing patients

Total score	Category	Management recommendations
0 Points	Common nevus	Reassessment every 1-2 years in community
1 Point	Low risk nevus	Non-urgent referral to ophthalmology clinic for imaging with long-term surveillance every 6-12 months depending on risk of malignancy
2 Points	High risk nevus	
≥ 3 Points	Probable melanoma	Urgent referral (within 2 weeks) to ophthalmology clinic

Adapted from: Damato BE. Can the MOLES acronym and scoring system improve the management of patients with melanocytic choroidal tumours? Eye (Lond). 2023;37(5):830-6.

Supplementary Table 3: TFSOM-DIM scoring system

**Risk factor**	**T**hickness (on US)	**F**luid subretinal (on OCT)	**S**ymptoms (on Snellen chart)	**O**range Pigment (on AF)	**M**elanoma hollow (on US)	**Diam**eter (on FP)
0 Points	≤ 2mm	No SRF	Visual acuity > 20/50	Absent	Internal acoustic density solid	≤ 5mm
1 Point	> 2mm	SRF-Cap or SRF ≤ 3mm from tumor	Visual acuity ≤ 20/50	Present	Internal acoustic density hollow	> 5mm

US, ultrasonography; OCT, optical coherence tomography; AF, fundus autofluorescence; FP, fundus photography; SRF, subretinal fluid

Adapted from Shields CL, Dalvin LA, Ancona-Lezama D, Yu MD, Di Nicola M, Williams BK, Jr., et al. CHOROIDAL NEVUS IMAGING FEATURES IN 3,806 CASES AND RISK FACTORS FOR TRANSFORMATION INTO MELANOMA IN 2,355 CASES: The 2020 Taylor R. Smith and Victor T. Curtin Lecture. Retina. 2019;39(10):1840-51.

Supplementary Table 4: Demographic data, MOLES categories and TFSOM-DIM scores for 695 nevi

		MOLES scores				
Feature	Category	0: Common nevus	1: Low risk nevus	2: High risk nevus	≥ 3: Probable melanoma	Total
Sex	Male	173 (39%)	62 (43%)	26 (32%)	7 (27%)	**268 (39%)**
	Female	271 (61%)	82 (57%)	55 (68%)	19 (73%)	**427 (61%)**
Laterality	Right	233 (52%)	75 (52%)	38 (47%)	10 (39%)	**356 (51%)**
	Left	211 (48%)	69 (48%)	43 (53%)	16 (61%)	**339 (49%)**
Location of nevus	Superior quadrant	89 (20%)	20 (14%)	14 (17%)	1 (4%)	**124 (18%)**
	Nasal quadrant	116 (26%)	39 (27%)	19 (24%)	6 (23%)	**180 (26%)**
	Inferior quadrant	62 (14%)	22 (15%)	14 (17%)	7 (27%)	**105 (15%)**
	Temporal quadrant	122 (28%)	51 (35%)	25 (31%)	10 (39%)	**208 (30%)**
	Macula	55 (12%)	12 (8%)	9 (11%)	2 (8%)	**78 (11%)**
Mushroom shape	Absent	444 (100%)	143 (99%)	80 (99%)	26 (100%)	**693 (99.7%)**
	Incipient	-	1 (1%)	1 (1%)	-	**2 (0.3%)**
	Present	-	-	-	-	**-**
Orange Pigment	Absent	444 (100%)	135 (94%)	59 (73%)	16 (62%)	**654 (94%)**
	Dusting	-	9 (6%)	18 (22%)	6 (23%)	**33 (5%)**
	Clumping	-	-	4 (5%)	4 (15%)	**8 (1%)**
Large size	Diameter < 3DD and thickness <1mm	444 (100%)	47 (33%)	22 (27%)	3 (12%)	**516 (74%)**
	Diameter 3-4DD and/or thickness 1-2mm	-	97 (67%)	20 (25%)	5 (19%)	**122 (18%)**
	Diameter >4DD and/or thickness >2mm	-	-	39 (48%)	18 (69%)	**57 (8%)**
Enlargement	None/ no baseline photography	444 (100%)	120 (83%)	63 (78%)	11 (42%)	**638 (92%)**
	Unsure growth/ New lesion without previous ophthalmoscopy	-	24 (17%)	12 (15%)	5 (19%)	**41 (6%)**
	Documented growth/ New confirmed tumor/ Diameter > 5DD or thickness >3mm	-	-	6 (7%)	10 (39%)	**16 (2%)**
Subretinal Fluid	Absent	444 (100%)	131 (91%)	68 (84%)	17 (65%)	**660 (95%)**
	SRF only on OCT	-	13 (9%)	13 (16%)	8 (31%)	**34 (4.9%)**
	SRF visible on ophthalmoscopy/ extending beyond tumor margins	-	-	-	1 (4%)	**1 (0.1%)**

TFSOM-DIM (5-year growth rate)	Total assessed	84	63	44	20	**211**
	0 (1%)	72 (86%)	31 (49%)	3 (7%)	-	**106 (50%)**
	1 (11%)	12 (14%)	26 (41%)	22 (50%)	7 (35%)	**67 (32%)**
	2 (22%)	-	6 (10%)	16 (37%)	8 (40%)	**30 (14%)**
	3 (34%)	-	-	3 (7%)	4 (20%)	**7 (3%)**
	4 (51%)	-	-	-	1 (5%)	**1 (1%)**
	5 (55%)	-	-	-	-	**-**
	6	-	-	-	-	**-**
Thickness	≤ 2mm	84 (100%)	63 (100%)	44 (100%)	17 (85%)	**208 (99%)**
	> 2mm	-	-	-	3 (15%)	**3 (1%)**
Fluid (subretinal)	Absent	84 (100%)	61 (97%)	37 (84%)	12 (60%)	**194 (92%)**
	Present	-	2 (3%)	7 (16%)	8 (40%)	**17 (8%)**
Symptoms	Vision ≥20/50	72 (86%)	52 (83%)	36 (82%)	19 (95%)	**179 (85%)**
	Vision ≤ 20/50	12 (14%)	11 (18%)	8 (18%)	1 (5%)	**32 (15%)**
Orange pigment	Absent	84 (100%)	62 (98%)	31 (71%)	12 (60%)	**189 (90%)**
	Present	-	1 (2%)	13 (29%)	8 (40%)	**22 (10%)**
Melanoma hollow on US	Solid	84 (100%)	60 (95%)	39 (89%)	15 (75%)	**198 (94%)**
	Hollow	-	3 (5%)	5 (11%)	5 (25%)	**13 (6%)**
Diameter	< 5mm	84 (100%)	42 (67%)	14 (32%)	6 (30%)	**146 (69%)**
	≥ 5mm	-	21 (33%)	30 (68%)	14 (70%)	**65 (31%)**
	**Total (MOLES)**	**444 (64%)**	**144 (21%)**	**81 (12%)**	**26 (4%)**	**695 (100%)**
	**Total (TFSOM-DIM)**	**84 (40%)**	**63 (30%)**	**44 (21%)**	**20 (10%)**	**211 (100%)**

DD, disc diameter; OCT, optical coherence tomography; SRF, subretinal fluid; US, ultrasound

Supplementary Table 5: Demographic data and MOLES categories for 53 melanoma

Feature	Category	MOLES scores<br>≥ 3:<br>Probable melanoma
MOLES scores	3	12 (23%)
	4	8 (15%)
	5	8 (15%)
	6	9 (17%)
	7	4 (8%)
	8	6 (11%)
	9	5 (9%)
	10	1 (2%)
Sex	Male	29 (55%)
	Female	24 (45%)
Laterality	Right	27 (51%)
	Left	26 (49%)
Location of melanoma	Superior quadrant	7 (13%)
	Nasal quadrant	15 (28%)
	Inferior quadrant	10 (19%)
	Temporal quadrant	19 (36%)
	Macula	1 (2%)
Mushroom shape	Absent	41 (77%)
	Incipient	4 (8%)
	Present	8 (15%)
Orange Pigment	Absent	21 (40%)
	Dusting	18 (34%)
	Clumping	14 (26%)
Large size	Diameter < 3DD and thickness <1mm	1 (2%)
	Diameter 3-4DD and/or thickness 1-2mm	15 (28%)
	Diameter >4DD and/or thickness >2mm	37 (70%)
Enlargement	None/ no baseline photography	13 (25%)
	Unsure growth/ New lesion without previous ophthalmoscopy	6 (11%)
	Documented growth/ New confirmed tumor/ Diameter > 5DD or thickness >3mm	34 (64%)
Subretinal Fluid	Absent	7 (13%)
	SRF only on OCT	28 (53%)
	SRF visible on ophthalmoscopy/ extending beyond tumor margins	18 (34%)
	**Total (MOLES)**	53 (100%)

DD, disc diameter; OCT, optical coherence tomography; SRF, subretinal fluid

Supplementary Table 6: TFSOM-DIM scores for 53 melanomas

Feature	Category	MOLES scores<br>≥ 3:<br>Probable melanoma
TFSOM-DIM (5-year growth rate)	Total assessed	36
	0 (1%)	-
	1 (11%)	-
	2 (22%)	2 (6%)
	3 (34%)	9 (25%)
	4 (51%)	9 (25%)
	5 (55%)	8 (22%)
	6	8 (22%)
Thickness	≤ 2mm	12 (33%)
	> 2mm	24 (67%)
Fluid (subretinal)	Absent	7 (19%)
	Present	29 (81%)
Symptoms	Vision ≥20/50	19 (53%)
	Vision ≤ 20/50	17 (47%)
Orange pigment	Absent	11 (31%)
	Present	25 (69%)
Melanoma hollow on US	Solid	3 (8%)
	Hollow	33 (92%)
Diameter	< 5mm	9 (25%)
	≥ 5mm	27 (75%)
	**Total (TFSOM-DIM)**	36 (100%)

US, ultrasonography
